# Fahr's disease presenting as Parkinson's disease along with dysphagia and dysarthria: A case report

**DOI:** 10.1002/ccr3.7358

**Published:** 2023-05-20

**Authors:** Dilip Sapkota, Srijana Neupane, Prashant Pant, Oshan Shrestha, Pragyat Singh, Diwas Sapkota

**Affiliations:** ^1^ Chormara Primary Health Care Centre Shivanagar Nepal; ^2^ Department of Internal Medicine Star Hospital Lalitpur Nepal; ^3^ College of Medicine Nepalese Army Institute of Health Sciences Kathmandu Nepal; ^4^ Department of Radiology Bharatpur Hospital Chitwan Nepal

**Keywords:** basal ganglia, Fahr's disease, idiopathic calcification, neurocognitive disorder

## Abstract

Fahr's disease, a rare motor and neurocognitive condition, is characterized by idiopathic calcification of basal ganglia. This article presents such case of 61‐year‐old female, exhibiting movement, speech, and swallowing difficulties with multiple calcifications in brain in NCCT. Early and supportive management can lead to improved outcomes and prevent unnecessary interventions.

## Graphical abstract

Image showing calcification over the basal ganglia which is characteristic of the Fahr's disease.

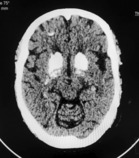

## 1 INTRODUCTION

Fahr's disease is a rare neurological disorder characterized by the presence of irregular calcium build up and consequent cell loss in specific regions of the brain, particularly the basal ganglia.^1^ Inheritance of Fahr's Disease is often observed in an autosomal dominant manner with incomplete and age‐related manifestation, although it can also be passed down as an autosomal recessive character or arise sporadically. Additionally, some research has suggested the presence of anticipation in this disorder.^2^ Patients with Fahr's disease commonly exhibit movement disorders, with parkinsonism being the most prevalent at 57%. Other movement disorders observed include chorea, tremor, dystonia, athetosis, and orofacial dyskinesia. Additional features include cognitive impairment, cerebellar signs, gait disorders, speech disorders, pyramidal signs, psychiatric symptoms, sensory changes, and pain.^3^ In this case report, we share case details of 61‐years‐old female who developed movement, speech, and swallowing difficulties and had features of multiple calcifications in NCCT brain.

This case report is in line with CARE guidelines.^4^

## 2 CASE REPORT

This case report presents a 61‐year‐old female, a resident of rural area, presented with complaints of movement and speech difficulty. The patient developed her first symptom 2 years ago which manifested as slow pace of walking, and over the period of 1 year, the symptoms gradually progressed and the existing symptoms were accompanied by tremors in her right hand which affected her ability to hold utensils and change clothes. In addition, the patient experienced frequent slippage of objects from her hands and faced slight difficulty in maintaining balance. Six months back from the day of the presentation, the patient started experiencing changes in her speech, characterized by slow and slurred speech that gradually worsened, making it difficult for others to understand. The patient also reported difficulty in swallowing her solid foods and feeling of getting stuck in her throat. The patient had been avoiding certain foods and eating in smaller portions. Swallowing difficulty gradually started to get accompanied by choking episodes more frequently, prompting her and her family to seek medical attention. The patient denied any mood and behavioral symptoms, memory loss, exposure to toxic substances, head trauma, seizures, weight loss, loss of consciousness, and cerebrovascular accidents. The patient mentioned that her father had a history of features of dementia in his early 80s. The patient has a medical history of type 2 diabetes mellitus and is currently under oral Hypoglycaemic therapy (Metformin, 850 mg, per oral, two times a day). There are no other medication history.

### 2.1 Timeline

**FIGURE 1:**
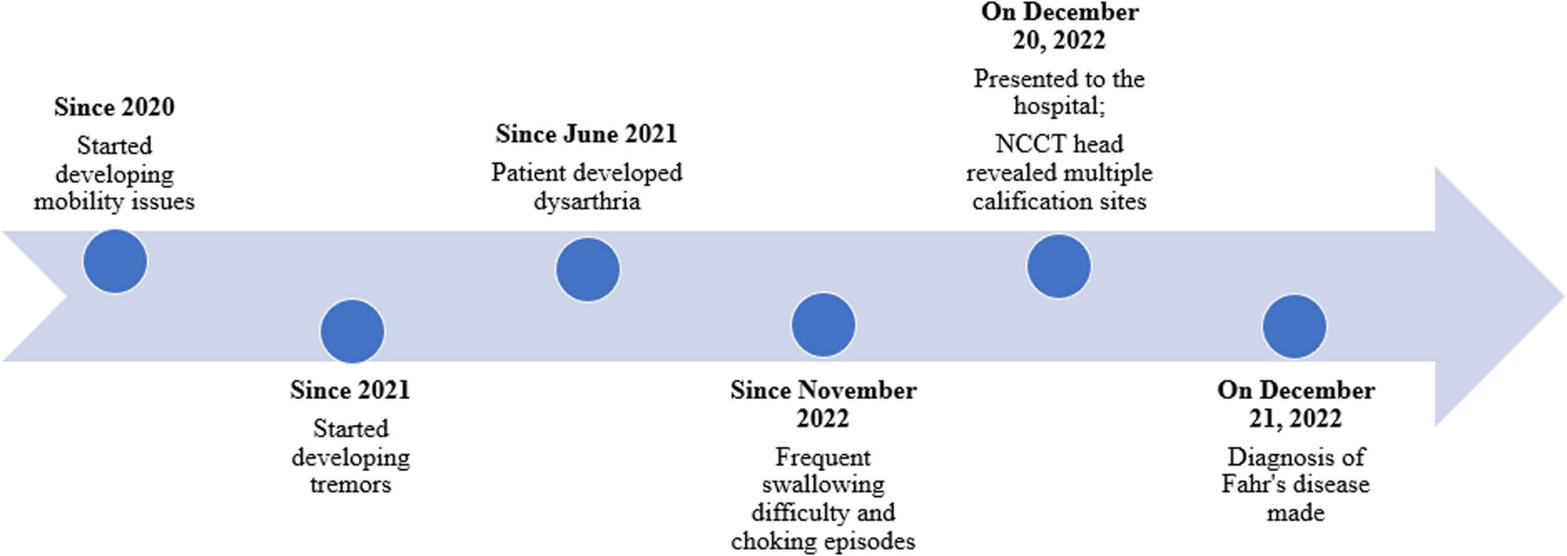
Timeline showing major events.

The patient's symptoms exhibited an insidious onset and a slow progression over a period of 2 years. The chronological sequence of major clinical events that occurred during this time is presented in the following timeline in Figure 1.

### 2.2 Diagnostic assessments

On the day of the presentation, physical examination revealed resting tremors of the right hand and bradykinesia in her all limbs. The patient had difficulty with motor tasks involving the right hand such as buttoning and unbuttoning clothes and picking objects. Her speech was slow, dysarthric, and difficult to interpret and comprehend. No evidence of spasticity and motor weakness or sensory abnormality was noted. She was conscious and oriented to time place and person and had normal neurocognitive functions. Reports of laboratory tests are presented in Table 1.

**TABLE 1:** Reports of laboratory tests.

Complete blood count	
Hemoglobin	13.1 g/dL
Red blood cell count	4.6 millions/mL
Total leucocyte count	9600/cm^3^
Differential count	(neutrophils: 62%; leucocytes: 30%; eosinophils: 6%; monocytes: 2%)
Platelets	236,000/cm^3^
Biochemistry	
Random blood sugar	140 mg/dL
Fasting blood sugar	126 mg/dL
HbA1c	7.5%
Total bilirubin	0.8 mg/dL
Alkaline phosphatase	140 μ/L
AST	28.2 μ/L
ALT	32.2 μ/L
Urea	15.6 mg/dL
Creatinine	0.8 mg/dL
Sodium	138.5 mEq/L
Potassium	4.1 mEq/L
Calcium	9.9 mg/dL
Phosphorus	3.8 mg/dL
Parathyroid Hormone (PTH)	36 pg/mL
Vitamin D (25‐hydroxy)	60 ng/mL
Urine Routine examination	
Albumin	Nil
Glucose	Nil
Pus cells	2–3/high power field
Red blood cells	Nil
Epithelial cells	2–4/high power field
Casts	Nil

**FIGURE 2:**
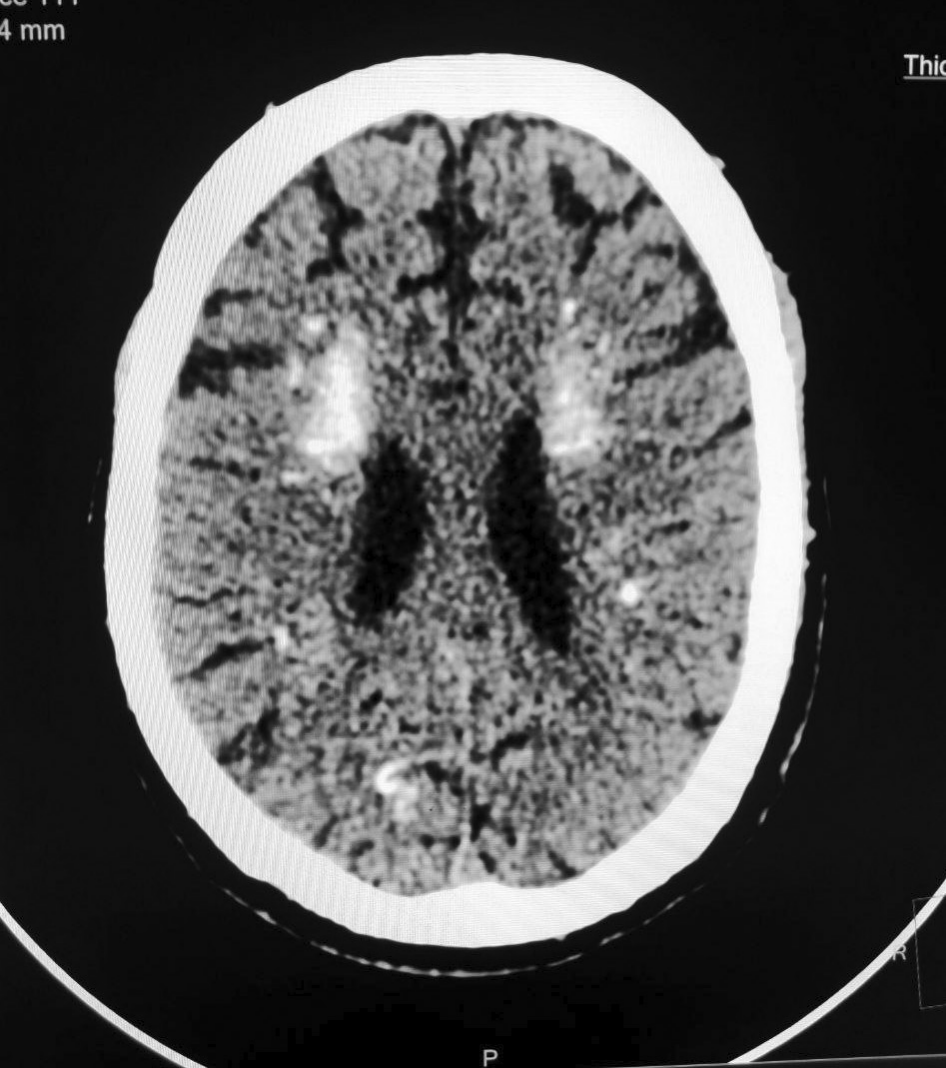
Image showing white matter calcification.

**FIGURE 3:**
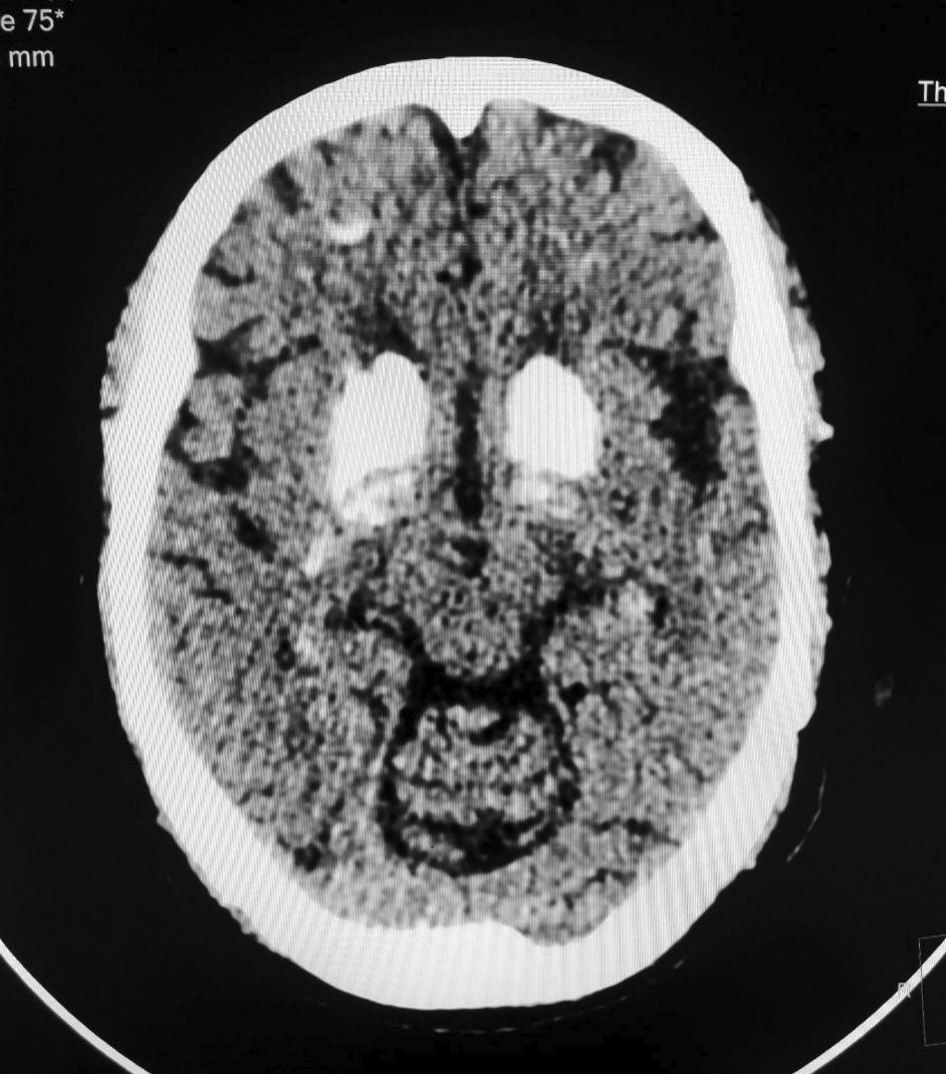
Image showing calcification over basal ganglia.

**FIGURE 4:**
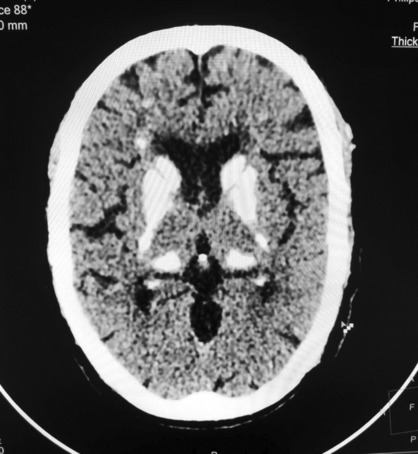
Image showing calcification over thalamus.

**FIGURE 5:**
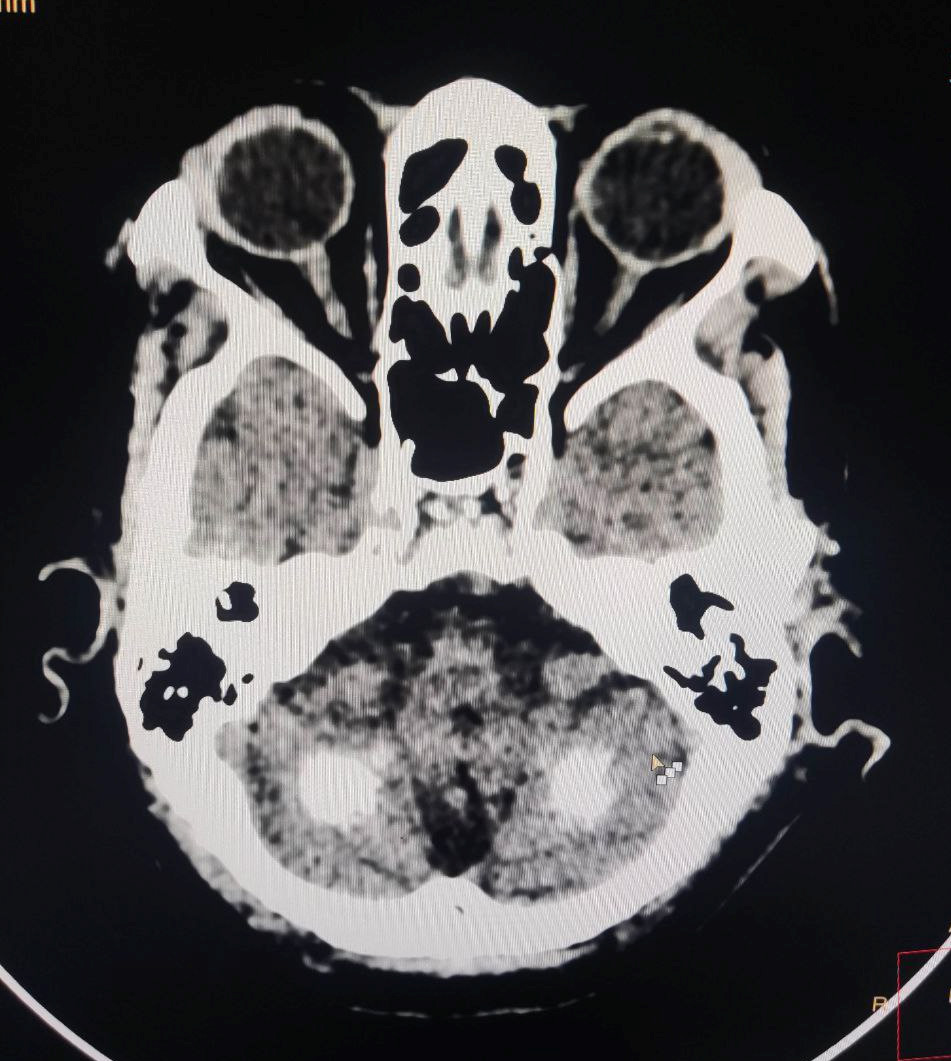
Image showing calcification over dentate nuclei.

The Non‐Contrast CT (NCCT) of the head revealed the presence of multiple calcifications in both cerebral hemispheres. These calcifications were symmetrically distributed and affected various regions of the brain, including the white matter (Figure 2), basal ganglia (specifically, the caudate and lentiform nuclei) (Figure 3), the bilateral thalami (Figure 4), and the dentate nuclei (Figure 5). Additionally, the imaging also indicated the presence of calcifications in the bilateral subcortical white matter. Notably, there was no mass effect, brain edema, or signs of parenchymal atrophy.

Considering the history, examinations, and the NCCT features, the patient was diagnosed to have Fahr's disease with predominant Parkinsons' symptoms.

### 2.3 Treatment

The patient after being diagnosed of having Fahr's disease with predominant Parkinson's symptoms, she was prescribed levodopa‐carbidopa (100gmg + 10 mg), per oral, two times a day. Patient was initially prescribed for 1 month after which review was done. Additionally, the patient was recommended to undergo physical therapy twice a week to enhance her mobility, and speech therapy once a week to address speech and swallowing difficulties. Genetic counseling was also offered to the patient and her family to aware them of the disease's inheritance pattern.

### 2.4 Follow‐up

The patient is currently under treatment. One‐month follow‐up has shown mild improvement in bradykinesia and tremors. At the follow‐up after 1 month, patient was prescribed with the same medications for next 3 months and was called for follow‐up for review at the end of 3 months. The patient is in regular follow‐up and monitoring to make any necessary adjustments in her treatment plan.

## 3 DISCUSSION

Fahr's disease is also termed bilateral strio‐pallido‐dentate calcinosis or primary familial brain calcification (PFBC) or calcinosis nucleorum.^5^ A bilateral, symmetrical, intra‐cranial calcification characterizes Fahr's disease with a predilection for the basal ganglia and the dentate nuclei. Because of the symmetrical involvement of these nuclei, descriptive terminology, BSPDC, has been put forth.^6^ Genetic alterations have been attributed to genes in the region of chromosome 14.^7^ These conditions predominantly involve the basal ganglia. The condition that has been closely described with diffuse, bilateral, symmetric striopallidodentate calcinosis is primary hypoparathyroidism.^8^, ^9^ Other causes include lupus, tuberous sclerosis, Alzheimer's disease, myotonic muscular dystrophy, and mitochondrial encephalopathies.^10^ The patient presented in the case has no underlying cause and no known family history of the condition.

The most common neurological manifestations include headaches, seizures, and movement disorders. Other specific manifestations include gait disturbances, dystonia, paresis, speech alterations, dementia, Parkinsonism, tremors, and chorea.^11^ Movement disorders accounted for 55% of the total symptomatic patients. Of the movement disorders, parkinsonism accounted for 57%, chorea 19%, tremor 8%, dystonia 8%, athetosis 5%, and orofacial dyskinesia 3%. Overlap of signs referable to different areas of the central nervous system (CNS) was common. Other neurologic manifestations included: cognitive impairment, cerebellar signs, pyramidal signs, psychiatric features, sensory changes, orthostatic hypotension, and pain.^12^ In our case, the patient presented with Parkinson's symptoms along with dysarthria and dysarthria. These symptoms of dysphagia and dysarthria can be particularly distressing for patients with neurological disorders, as they can lead to social isolation and difficulties with everyday activities such as eating and communicating. Dysphagia, or difficulty with swallowing, can also lead to a risk of aspiration pneumonia, which is a common complication in patients with neurodegenerative disorders such as Parkinson's disease and progressive supranuclear palsy (PSP).

The clinical diagnosis of Fahr's disease is based on the combination of clinical features, brain imaging, and the exclusion of other causes of intracranial calcification.^8^ To differentiate Fahr's disease from Fahr's syndrome, laboratory examinations should include tests for blood calcium and parathormone in addition to the other routine blood tests. There is one case report with late‐onset Fahr's disease and basal ganglia calcification.^13^ The publication of this case has brought Fahr's disease into focus as a possible diagnosis that should be taken into account when examining older patients with cognitive impairment, like Alzheimer's, and movement disorders, such as Parkinson's.

## 4 CONCLUSION

Fahr's disease can exhibit clinical features that may overlap with Parkinson's disease, including dysphagia. Diagnosis of this condition can be challenging, particularly in elderly individuals, and delayed detection can lead to significant physical, cognitive, and social impairments. Therefore, it is crucial to investigate any neurological symptoms and perform appropriate brain CT imaging to rule out Fahr's disease. Although there is currently no definitive treatment for this disorder, early and supportive management can lead to improved outcomes and prevent unnecessary interventions throughout the disease course.

## AUTHOR CONTRIBUTIONS

**Dilip Sapkota:** Conceptualization; data curation; project administration; validation; writing – original draft; writing – review and editing. **Srijana Neupane:** Conceptualization; data curation; project administration; validation; writing – original draft; writing – review and editing. **Prashant Pant:** Conceptualization; data curation; project administration; validation; writing – review and editing. **Oshan Shrestha:** Conceptualization; project administration; validation; writing – original draft; writing – review and editing. **Pragyat Singh:** Data curation; project administration; validation; writing – review and editing. **Diwas Sapkota:** Project administration; supervision; validation; writing – review and editing.

## FUNDING INFORMATION

This article did not receive any grants.

## CONFLICT OF INTEREST STATEMENT

The authors declare that there is no conflict of interests.

## CONSENT

Written informed consent was obtained from the patient for publication of this case report and accompanying images. A copy of the written consent is available for review by the Editor‐in‐Chief of this journal on request.

## PATIENT PERSPECTIVE

The patient was distressed due to her deteriorating quality of life. Condition. The patient and patient‐party were counseled. Some improvements in symptoms has brought positive outlook in the patient and her family.

## Data Availability

All the findings are present within the manuscript.
